# Explore the changes of metabolites in feces and serum of acute pancreatitis patients with different etiologies by LC-MS based metabolomics strategy

**DOI:** 10.3389/fphar.2025.1614713

**Published:** 2025-06-25

**Authors:** Meng-Yan Cui, Miao-Yan Fan, Jun Wu, Su-Min Chen, Meng-Qi Zhao, Qiao-Li Jiang, Jing-Jing Wang, Yue Zeng, Yu Zhao, Ying-Ying Lu

**Affiliations:** ^1^ Department of Gastroenterology, Jiading branch of Shanghai General Hospital, Shanghai Jiao Tong University School of Medicine, Shanghai, China; ^2^ Department of Gastroenterology, Shanghai General Hospital, Shanghai Jiao Tong University School of Medicine, Shanghai, China; ^3^ Shanghai Key Laboratory of Pancreatic Disease, Shanghai Jiao Tong University School of Medicine, Shanghai, China; ^4^ Department of Clinical laboratory, Jiading branch of Shanghai General Hospital, Shanghai Jiao Tong University School of Medicine, Shanghai, China

**Keywords:** acute pancreatitis, biliary acute pancreatitis, hyperlipidemic acute pancreatitis, LC-MS, fecal metabolome, serum metabolome

## Abstract

**Background:**

Acute pancreatitis (AP) is a common abdominal inflammatory disease, which is mainly caused by gallstones, hyperlipidemia, and so on. Previous studies have explored the changes of serum metabolites in patients with AP. However, whether different etiologies have distinct impacts on the fecal and serum metabolites of the AP patients is still in suspense.

**Aim:**

This investigation was designed with dual objectives: (1) to systematically delineate etiology-specific alterations in metabolic profiles and associated pathway perturbations in AP cohorts, and (2) to evaluate their potential as diagnostic biomarkers and pathogenesis-targeted therapeutic strategies.

**Methods:**

Fifteen stool samples and fifteen serum samples from the patients with biliary acute pancreatitis (BAP) and hyperlipidemic acute pancreatitis (HAP), respectively, were analyzed. Metabolites were quantified by using ultrahigh performance liquid chromatography-mass spectrometry (UPLC-MS). The anti-inflammatory properties of Gibberellin A4 (GA4) and catechin were verified by RT-qPCR *in vitro*.

**Results:**

The metabolites in feces and serum of the AP group were significantly different from that of the control group. Compared with the control group, a total of 10 fecal metabolites were significantly altered in the AP group. In the serum metabolites, five differential metabolites were identified between the AP and control groups. Receiver operating characteristic analysis of the subjects found that important differential metabolites can distinguish AP patients with different etiology from healthy people, and *in vitro* experiments found that GA4 and catechin could reduce the expression of inflammatory factors in pancreatic 266-6 cell line.

**Conclusion:**

The significant differential metabolites between AP patients and healthy people can clearly distinguish the two groups, and GA4 and catechin have anti-inflammatory effects on pancreatic cell lines. The identification of distinct metabolites enabled to distinguish the AP patients with different etiologies.

## 1 Introduction

Acute pancreatitis (AP) is a common abdominal inflammatory disease that is associated with serious suffering and morbidity. Globally, the incidence of AP is increasing year by year (Spanier et al., 2013; Peery et al., 2019; Peery et al., 2015), which has become about 30–40 cases per 100,000 people per year (Petrov and Yadav, 2019). Despite the fact that the majority of AP patients present with mild illness, about 20%–25% of AP patients develop into critical illness, characterized by peripancreatic tissue and other distant organ dysfunction, even developing into multiple organ dysfunction syndrome (MODS), which is also the leading cause of AP-related death (Jin et al., 2019). Therefore, it is urgent to explore the pathogenesis and therapeutic targets of AP.

There are numerous etiologies of AP. Biliary acute pancreatitis (BAP) and hyperlipidemic acute pancreatitis (HAP) account for 70% of patients diagnosed with AP (Jin et al., 2019). Transient obstruction of the pancreatic duct by migrating gallstones is the most common cause (Lankisch et al., 2015). Exposure of the pancreas to biliary constituents can cause pancreatic acinar tissue damage. Besides, hypertriglyceridemia has become the second leading cause in China (Mukherjee et al., 2019). Pancreatic lipase hydrolyses excessing triglyceride and the free fatty acid accumulation jointly induce acinar cell and pancreatic capillary injury (Lin et al., 2022). Consequently, the pathogenesis of AP with different etiologies are different from each other. However, whether different etiologies have distinct impacts on the fecal and serum metabolites of the AP patients is still in suspense.

Metabolomics has been applied in the discovery of AP related biomarkers. Lankes et al. through liquid chromatography tandem mass spectrometry (LC-MS/MS) found that albumin and fibrinogen provided the potential to diagnose pancreatitis more specifically than pancreatic enzymes over a longer time period for its longer circulating half-lives (Lankes et al., 2015). Lusczek et al. through nuclear magnetic resonance (NMR) spectroscopy compared urinary metabolic profiles of patients with AP to healthy controls. They found that the significant increase in urinary adenosine and decrease in urinary citrate may reflect the patients’ inflammatory state (Lusczek et al., 2013). Hence, previous studies have shown that there are metabolic disorders in AP patients. The related metabolites in serum and urine have the potential to diagnose AP and, to some extent, reflect the inflammation of the body. However, there were no reports about fecal metabolites in AP patients in previous studies. Furthermore, whether different etiologies have different effects on the fecal and serum metabolism of the AP patients is still in suspense. And the correlation of differential metabolites in fecal and serum with clinical indicators remains unclear.

In this study, we explored the changes of fecal and serum metabolites and metabolic pathways in the AP patients with different etiologies. Furthermore, we explored the correlation of the relevant differential metabolites with clinical indicators. And investigated the impact of blood-borne fecal metabolites on pancreatic acinar cells.

## 2 Materials and methods

### 2.1 Study design

This study was designed to explore the changes of fecal and serum metabolites and metabolic pathways in AP patients with different etiologies. This prospective study, which was approved by the Institutional Review Board of Shanghai General Hospital, collected samples from July 2022 to July 2023. Written informed consent was obtained from all individuals before enrollment. All the authors had access to the study data and had reviewed and approved the final manuscript.

### 2.2 Study population

Patients with AP were recruited in inpatient department of the Shanghai General Hospital. Only patients who met the Revised Atlanta Classification (RAC) criteria were included in this study. The diagnosis of AP met two of the following three features: (1) abdominal pain consistent with acute pancreatitis (acute onset of a persistent, severe, epigastric pain often radiating to the back); (2) serum lipase activity (or amylase activity) at least three times greater than the upper limit of normal; and (3) characteristic findings of acute pancreatitis on contrast-enhanced computed tomography (CECT) and less commonly magnetic resonance imaging (MRI) or transabdominal ultrasonography (Banks et al., 2013).

Inclusion criteria for BAP met the following features: meeting the diagnostic criteria for AP; (4) gallbladder stones confirmed by ultrasound or MRCP (van Geenen et al., 2010).

Inclusion criteria for HAP met the following features: meeting the diagnostic criteria for AP; (4) the level of triglyceride (TG) in serum reached or exceeded 11.3 mmol/L; (5) excluding other causes, such as alcoholic, gallbladder, etc. and the TG was between 5.65 and 11.3 mmol/L (Banks et al., 2013).

Healthy controls were recruited from health management center of Jiading branch of Shanghai General Hospital. They had never been diagnosed with severe digestive disorders, such as acute or chronic pancreatitis, cholecystitis or cholangitis, and hepatic insufficiency.

### 2.3 Sample collection

Fecal samples were collected in sterile fecal collection containers from each participant. The samples were immediately frozen at −80°C until LC-MS analysis.

All the AP patients took blood on the second day of admission to the hospital. Healthy controls were fasted for 12 h before blood collection to rule out the effects of food or alcohol. Blood was collected in 1.5 mL EP tubes without anticoagulant. After centrifugation, serum was subsampled (300 uL) into prepared sterile tubes and frozen at −80°C until LC-MS analysis.

### 2.4 LC-MS analysis

The LC-MS/MS analysis of sample was conducted on a Thermo UHPLC-Q Exactive system equipped with an ACQUITY HSS T3 column (100 mm × 2.1 mm i.d., 1.8 μm; Waters, United States) at Majorbio Bio-Pharm Technology Co. Ltd. (Shanghai, China). The mobile phases consisted of 0.1% formic acid in water: acetonitrile (95:5, v/v) (solvent A) and 0.1% formic acid inacetonitrile: isopropanol: water (47.5:47.5, v/v) (solvent B). The flow rate was 0.40 mL/min and the column temperature was 40°C.

The UPLC system was coupled to a Thermo UHPLC-Q Exactive Mass Spectrometer equipped with an electrospray ionization (ESI) source operating in positive mode and negative mode. The optimal conditions were set as followed: source temperature at 400°C; sheath gas flow rate at 40 arb; Aux gas flow rate at 10 arb; ion-spray voltage floating (ISVF) at −2800 V in negative mode and 3500 V in positive mode, respectively; Normalized collision energy, 20-40-60 V rolling for MS/MS. Full MS resolution was 70,000, and MS/MS resolution was 17,500. Data acquisition was performed with the Data Dependent Acquisition (DDA) mode. The detection was carried out over a mass range of 70–1,050 m/z.

### 2.5 Metabolomics data analysis and identification

The pretreatment of LC/MS raw data was performed by Progenesis QI (Waters Corporation, Milford, United States) software, and a three-dimensional data matrix in CSV format was exported. The information in this three-dimensional matrix included: sample information, metabolite name and mass spectral response intensity. Internal standard peaks, as well as any known false positive peaks (including noise, column bleed, and derivatized reagent peaks), were removed from the data matrix, deredundant and peak pooled. At the same time, the metabolites were identified by searching database, and the main databases were the HMDB (http://www.hmdb.ca/), Metlin (https://metlin.scripps.edu/) and Majorbio Database.

The data were analyzed through the free online platform of majorbio choud platform (cloud.majorbio.com). Metabolic features detected at least 80% in any set of samples were retained. After filtering, minimum metabolite values were imputed for specific samples in which the metabolite levels fell below the lower limit of quantitation, and each Metabolic features were normalized by sum. To reduce the errors caused by sample preparation and instrument instability, the response intensity of the sample mass spectrum peaks was normalized by the sum normalization method, and then the normalized data matrix was obtained. Meanwhile, variables with relative standard deviation (RSD) > 30% of QC samples were removed, and log10 processing was performed to obtain the final data matrix for subsequent analysis.

### 2.6 Cell culture

The 266-6 (CTCC-007-0526) cultured in Dulbecco’s modified Eagle’s medium (DMEM) supplemented with 5% heat-inactivated fetal bovine serum (FBS), 1% penicillin and streptomycin and 0.1% *Mycoplasma* Removal Agent (immocell, China) at 37°C, 95% humidity, and 5% CO_2_. Both DMEM and FBS were obtained from Bioagrio, china.

### 2.7 Cell viability

The effects of LPS (Sigma-Aldrich, United States), Gibberellin A4 and Catechin (MedChemExpress, China) on the cell viability of 266–6 cells were measured using CCK8 (Topscience, China) according to the manufacturer’s instructions. 266-6 cells were incubated at different concentrations of Gibberellin A4 (1 μM, 5 μM, 10 μM, 20 μM, 30 μM) or Catechin (0.1 μM, 1 μM, 10 μM, 50 μM, 100 μM) for 24 h. Another group of cells were incubated at 1 μg/mL LPS (Sigma-Aldrich, United States) and Gibberellin A4 or Catechin. Then 10 μL CCK eight reagent was added to the cells, followed by another 2 h of incubation. Finally, the absorbance at 450 nm was measured.

### 2.8 Real-time quantitative PCR (RT-qPCR) analysis

The inflammatory cytokines were quantified by real-time PCR. Reverse transcription (RT) was conducted using HyperScript III RT SuperMix for qPCR in conjunction with the gDNA Remover Kit (both from EnzyArtisan, Shanghai, China). All primers, which were customsynthesized by EnzyArtisan (China), are listed in Supplementary Table S1. Real-time PCR was performed with 2xS6 Universal SYBR qPCR Mix (EnzyArtisan, Shanghai, China), adhering strictly to the manufacturer’s guidelines. The relative expression levels of the genes were determined using the 2^−ΔΔCT^ method.

### 2.9 Statistical analysis

Perform variance analysis on the matrix file after data preprocessing. The R package “ropls” (Version 1.6.2) was used to perform principal component analysis (PCA) and orthogonal least partial squares discriminant analysis (OPLS-DA), and 7-cycle interactive validation evaluating the stability of the model. The metabolites with VIP > 1, p < 0.05 were determined as significantly different metabolites based on the Variable importance in the projeciton (VIP) obtained by the OPLS-DA model and the p-value generated by Student’s t-test.

Differential metabolites among two groups were mapped into their biochemical pathways through metabolic enrichment and pathway analysis based on KEGG database (http://www.genome.jp/kegg/). These metabolites could be classified according to the pathways they involved or the functions they performed. Enrichment analysis was used to analyze a group of metabolites in a function node whether appears or not. The principle was that the annotation analysis of a single metabolite develops into an annotation analysis of a group of metabolites. Python packages “scipy.stats” (https://docs.scipy.org/doc/scipy/) was used to perform enrichment analysis to obtain the most relevant biological pathways.

Pearson correlation analysis was performed between differential metabolites and clinical indicators. Receiver operating characteristic (ROC) was performed for differential metabolites associated with clinical indicators.

## 3 Results

### 3.1 Characteristics of study subjects

The demographic and clinical characteristics for 30 AP participants and 20 controls were presented in Table 1. No statistically significant difference in age, gender, and the proportion of hypertension was found (*P* > 0.05) between the AP group and the control group. Body mass index (BMI) and diabetes prevalence were found to differ between the AP and control groups (0.05 < *P* < 0.1).

**TABLE 1:** Clinical characteristics of the AP and control groups.

Variable	AP	CON	t/Z	*P* value
n	30	20	-	-
Age (years, mean ± SD)	51.20 ± 12.89	46.20 ± 12.06	1.379	0.174
Gender			1.797	0.180
Male (n, %)	22 (73.33)	11 (55.00)	-	-
Female (n, %)	8 (26.67)	9 (45.00)	-	-
BMI (kg/m^2^, mean ± SD)	26.36 ± 3.37	24.64 ± 3.05	1.828	0.074
Hypertension (n, %)	9 (30.00)	4 (20.00)	0.624	0.430
Diabetes (n, %)	12 (40.00)	3 (15.00)	3.571	0.059

The demographic and clinical characteristics for 15 BAP participants and 15 HAP participants were presented in Table 2. There was no statistically significant difference observed in age, gender, onset time, hospital stay, CTSI score, white blood cell count, and neutrophil ratio between the two groups (*P* > 0.05). Nevertheless, the level of the inflammatory indicators, such as C-reaction protein (CRP) and IL-6, were higher in the HAP group (*P* < 0.1). Additionally, it was shown that the onset age of HAP was younger than that of BAP (*P* < 0.05). The daily habits and customs of patients diagnosed with HAP were found to be comparatively inferior to those of patients diagnosed with BAP. This discrepancy was shown in terms of a higher rate of smoking (*P* < 0.05) and drinking (0.05 < *P* < 0.1) among the HAP patients. The rate of diabetes was also higher in the HAP group (*P* < 0.05).

**TABLE 2:** Clinical characteristics of the BAP and HAP groups.

Variable	BAP	HAP	t/Z	*P* value
n	15	15	-	-
Age (years, mean ± SD)	60.73 ± 10.53	41.67 ± 6.18	6.046	<0.001
Gender			0.682	0.409
Male (n, %)	10 (66.67)	12 (80.00)	-	-
Female (n, %)	5 (33.33)	3 (20.00)	-	-
BMI	25.25 ± 2.30	27.46 ± 3.96	−1.873	0.072
Hypertension (n, %)	5 (33.33)	4 (26.67)	0.159	0.690
Diabetes (n, %)	3 (20.00)	9 (60.00)	5.000	0.025
History of smoking (n, %)	2 (13.33)	8 (53.33)	5.400	0.020
History of drinking (n, %)	1 (6.66)	5 (33.33)	3.333	0.068
Onset time (day, mean ± SD)	1.93 ± 1.10	2.07 ± 1.22	−0.314	0.756
Hospital stay (day, mean ± SD)	7.27 ± 3.67	9.27 ± 6.26	−1.067	0.295
CTSI score (mean ± SD)	2.27 ± 1.79	3.00 ± 1.25	−1.299	0.205
White blood cell count (*10^9, mean ± SD)	9.90 ± 2.75	10.24 ± 3.20	−0.312	0.757
Neutrophil ratio (%, mean ± SD)	7.61 ± 2.68	7.91 ± 3.08	−0.288	0.775
C-reaction protein (mg/L, mean ± SD)	73.98 ± 56.50	125.29 ± 60.35	−2.404	0.023
IL-6 (pg/mL)	37.09 ± 39.00	77.07 ± 71.74	−1.896	0.068

### 3.2 Significant differences in overall structure of fecal metabolites in patients with AP of different etiologies compared with controls

**FIGURE 1:**
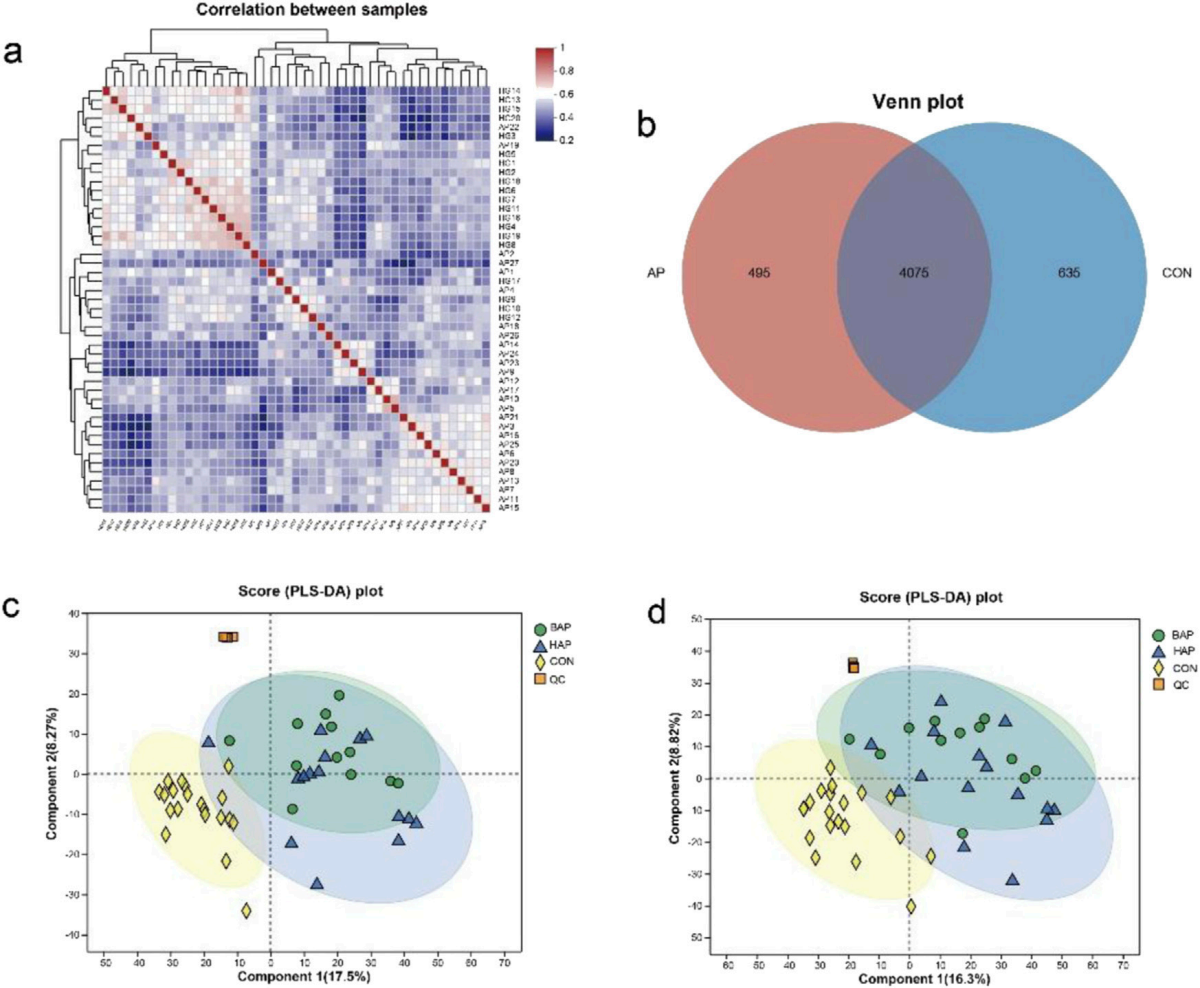
Differentiation of fecal metabolome samples from patients with AP and controls. **(a)** Correlation analysis of fecal samples between AP group and control group. **(b)** Venn analysis of fecal metabolites between AP group and control group. **(c)** PLS-DA of fecal metabolites in comparisons of patients with AP of different etiologies (positive ion). **(d)** PLS-DA of fecal metabolites in comparisons of patients with AP of different etiologies (negative ion).

Pearson correlation analysis showed that the fecal samples of AP group and control group were significantly separated (Figure 1a). As shown in Venn plot, 495 fecal metabolites were unique in AP patients, and 635 fecal metabolites were found in healthy people but not detected in AP patients (Figure 1b).

Partial least squares discriminant analysis (PLS-DA) recognition model was generated for the whole dataset to evaluate the clustering trend of the samples. The separation between AP and control group could be clearly observed in the PLS-DA patterns in both negative- and positive-ion modes, which demonstrated that there was a severe metabolic disorder in the patients diagnosed with AP. Besides, the BAP group and the HAP group were also significantly different from the control group. Nevertheless, there was a considerable degree of overlap in the fecal metabolites between the BAP group and HAP groups, suggesting that the distinction was not significant between the two groups. (Figures 1c,d). All of the analyses in the OPLS-DA pattern were consistent with those in the PLS-DA pattern (Supplementary Figure S1).

### 3.3 Differential metabolites and differential metabolic pathways in feces between the patients diagnosed with acute pancreatitis of different etiologies and the control group

**FIGURE 2:**
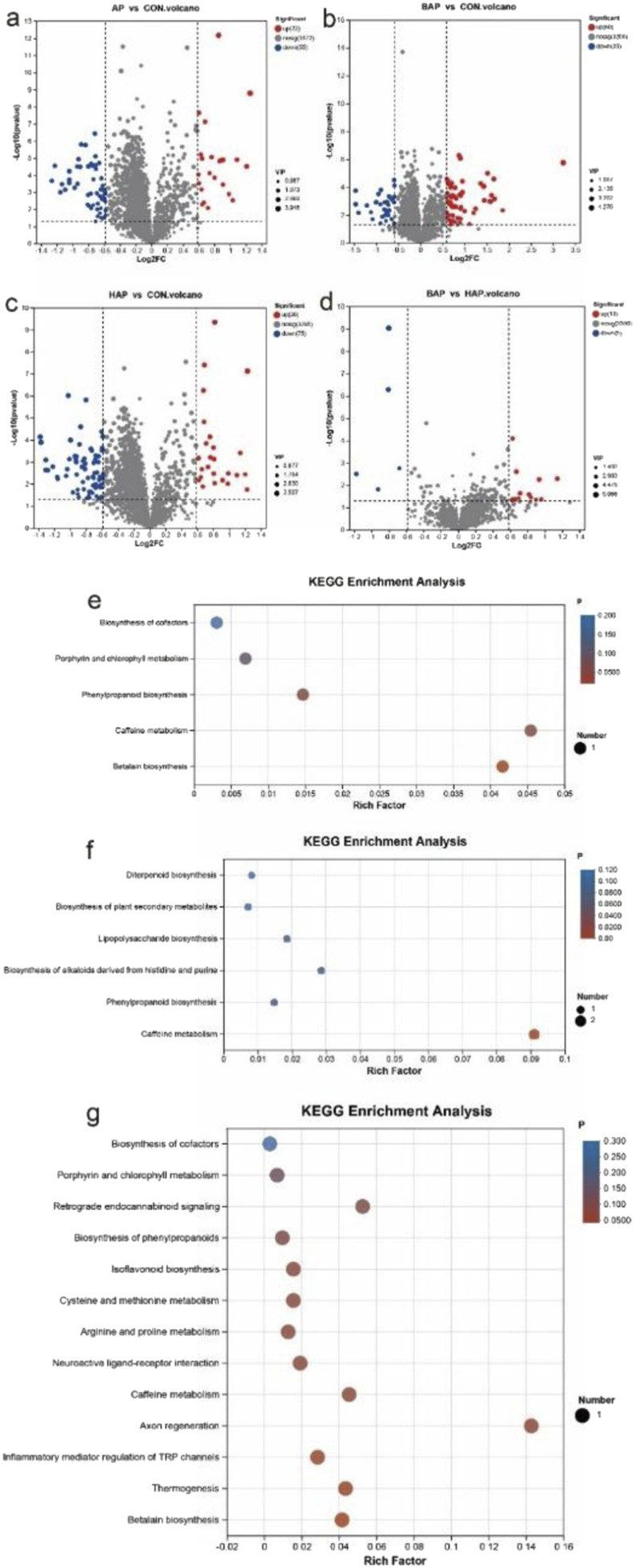
Analysis of fecal differential metabolites and metabolic pathways in patients with AP of different etiologies and control group. **(a)** Volcano map of differential metabolites in the AP and control groups. **(b)** Volcano map of differential metabolites in the BAP and control groups. **(c)** Volcano map of differential metabolites in the HAP and control groups. **(d)** Volcano map of differential metabolites in the BAP and HAP groups. **(e)** Metabolic pathway analysis of the differential metabolites between the AP and control groups. **(f)** Metabolic pathway analysis of the differential metabolites between the BAP and control groups. **(g)** Metabolic pathway analysis of the differential metabolites between the HAP and control groups.

Differential metabolites were identified by metabolites-expression-level analysis with *P* values ≤0.05 and fold change values ≥1.5, and VIP pred OPLS-DA>1. As shown in Figure 2a, 77 (22 upregulated and 55 downregulated) fecal differential metabolites were identified between the AP and control groups. Further screening for the metabolites with fold change values ≥2 and excluding related drug metabolism factors found that two metabolites were upregulated, and eight metabolites were downregulated in the AP group (Table 3). To further investigate the metabolic changes in the patients diagnosed with AP, metabolic pathway analysis was performed. As shown in Figure 2e, Cyclo-dopa 5-O-glucoside, significantly declined in the AP group, is a constituent of the betalain biosynthetic pathway (*P* < 0.05). Similarly, the decreased 3-Methylxanthine in the AP group is an essential component in caffeine metabolism (0.05 < *P* < 0.1). Sinapoyl Malate, which was considerably higher in the AP group, is a component of the phenylpropanoid biosynthesis pathway (0.05 < *P* < 0.1).

**TABLE 3:** Differentially expressed metabolites between AP and controls.

Metabolite	Predicted molecular pathways or biological functions	AP versus CON			AP
		VIP	*P*	FC (AP/CON)	
ESI+					
Methionyl Glutamate	-	2.7216	<0.0001	0.4268	↓^2^
GRK2 Inhibitor	-	3.9464	<0.0001	2.3813	↑^1^
1-Nonanol	-	2.7701	0.0003	0.4547	↓^9^
ESI-					
11-Hydroxy-9-tridecenoic acid	-	2.8167	0.0002	0.4851	↓^4^
3-(3,5-dihydroxyphenyl)-1-propanoic acid sulphate	-	2.8252	0.0010	0.4481	↓^8^
3-Methylxanthine	Caffeine metabolism	2.7593	0.0002	0.4160	↓^5^
Cyclo-dopa 5-O-glucoside	Betalain biosynthesis	2.7434	0.0003	0.4849	↓^6^
Sinapoyl Malate	Phenylpropanoid biosynthesis	2.4554	0.0029	2.0441	↑^10^
N1-(5-Phospho-a-D-ribosyl)-5,6-dimethylbenzimidazole	Porphyrin and chlorophyll metabolism; Biosynthesis of cofactors	2.7229	0.0007	0.4675	↓^7^
Catechin	-	3.2182	<0.0001	0.4850	↓^3^

^n^The order in which changes are evaluated based on VIP.

As shown in Figure 2b, 95 (60 upregulated and 35 downregulated) fecal differential metabolites were identified between the BAP and control groups. Further screening out the metabolites of fold change values ≥ 2 and excluding related drug metabolism factors found that seven metabolites were upregulated and seven metabolites were downregulated in the BAP group (Table 4). Subsequent metabolic pathway analysis revealed that 3-Methylxanthine and theobromine are constituents of the caffeine metabolism, with statistical significance (*P* < 0.05). Theobromine is a component found within the biosynthesis of alkaloids derived from histidine and purine (0.05 < *P* < 0.1). Keto-3-deoxy-D-manno-octulosonic acid, which was decreased in the BAP group, is a constituent of lipopolysaccharide biosynthesis (0.05 < *P* < 0.1). And sinapoyl malate, which was considerably higher in the BAP group, is a component of the phenylpropanoid biosynthesis (0.05 < *P* < 0.1) (Figure 2f).

**TABLE 4:** Differentially expressed metabolites between BAP and controls.

Metabolite	Predicted molecular pathways or biological functions	BAP versus CON			BAP
		VIP	*P*	FC (AP/CON)	
ESI+					
Phenylalanylhistidine	-	3.0892	0.0003	3.0472	↑^3^
Methionyl Glutamate	-	3.0740	0.0002	0.3616	↓^2^
Blumenol C O-[apiosyl-(1->6)-glucoside]	-	2.6404	0.0070	2.2987	↑^13^
Theobromine	Caffeine metabolism; Biosynthesis of alkaloids derived from histidine and purine	3.4335	<0.0001	2.8624	↑^1^
Gibberellin A7	Diterpenoid biosynthesis	2.4957	0.0045	3.6240	↑^10^
1-Nonanol	-	2.1754	0.0063	0.4595	↓^11^
ESI-					
11-Hydroxy-9-tridecenoic acid	-	2.9083	0.0021	0.4320	↓^8^
Aloesol	-	2.9335	0.0011	2.9586	↑^5^
Galbanic acid	-	2.8740	0.0035	2.3734	↑^9^
3-(3,5-dihydroxyphenyl)-1-propanoic acid sulphate	-	3.3065	0.0017	0.3620	↓^7^
3-Methylxanthine	Caffeine metabolism	2.7367	0.0068	0.3786	↓^12^
Sinapoyl Malate	Phenylpropanoid biosynthesis	3.1990	0.0011	2.9963	↑^4^
Benzyl sulfate	-	2.0246	0.0201	0.4718	↓^14^
Keto-3-deoxy-D-manno-octulosonic acid	Lipopolysaccharide biosynthesis	3.2598	0.0012	0.4985	↓^6^

^n^The order in which changes are evaluated based on VIP.

As shown in Figure 2c, 101 (26 upregulated and 75 downregulated) fecal differential metabolites were identified between the HAP and control groups. Further screening out the metabolites of fold change values ≥ 2 and excluding related drug metabolism factors found that four metabolites were upregulated and 10 metabolites were downregulated in the HAP group (Table 5). One of the differential metabolites, N-arachidonylethanolamine, which was increased in the HAP group, is involved in various metabolic processes, such as inflammatory mediator regulation of TRP channels, thermogenesis, axon regeneration, neuroactive ligand-receptor interaction, and retrograde endocannabinoid signaling (0.05 < *P* < 0.1). Besides, S-adenosyl-L-methioninamine, exhibited an elevated presence in the HAP group, is involved in the metabolism of cysteine, methionine, arginine, and proline (0.05 < *P* < 0.1). In addition, to the same as above, 3-Methylxanthine is a component of the metabolism of caffeine. It has been determined that cyclo-dopa 5-O-glucoside is a component of the betalain biosynthesis pathway (Figure 2g).

**TABLE 5:** Differentially expressed metabolites between HAP and controls.

Metabolite	Predicted molecular pathways or biological functions	HAP versus CON			HAP
		VIP	*P*	FC (AP/CON)	
ESI+					
(+)-Pisatin	Biosynthesis of phenylpropanoids; Isoflavonoid biosynthesis	2.6008	0.0024	0.4114	↓^8^
Methionyl-Glutamate	-	2.3391	0.0043	0.4826	↓^11^
Epidermin	-	3.2121	<0.0001	0.3848	↓^2^
1-Nonanol	-	3.507	<0.0001	2.351	↑^1^
GRK2 Inhibitor	-	2.6445	0.0016	0.4295	↓^5^
S-adenosyl-L-methioninamine	Cysteine and methionine metabolism; Arginine and proline metabolism	2.2121	0.0037	2.3038	↑^9^
ESI-					
Aloesol	-	2.4551	0.0040	2.1408	↑^10^
2-O-alpha-D-Galactopyranosyl-1-deoxynojirimycin	-	2.8924	0.0008	0.4042	↓^4^
3-Methylxanthine	Caffeine metabolism	2.5701	0.0048	0.4460	↓^12^
Cyclo-dopa 5-O-glucoside	Betalain biosynthesis	2.6326	0.0021	0.4873	↓^6^
N-arachidonylethanolamine	Inflammatory mediator regulation of TRP channels; Thermogenesis; Axon regeneration; Neuroactive ligand-receptor interaction; Retrograde endocannabinoid signaling	2.3677	0.0179	2.3406	↑^14^
N1-(5-Phospho-a-D-ribosyl)-5,6-di methylbenzimidazole	Porphyrin and chlorophyll metabolism; Biosynthesis of cofactors	2.7622	0.0023	0.4037	↓^7^
Pentadecenoic acid	-	2.4061	0.0059	0.4485	↓^13^
Catechin	-	3.4815	0.0001	0.3871	↓^3^

^n^The order in which changes are evaluated based on VIP.

As shown in Figure 2d, 18 (13 upregulated and five downregulated) fecal differences metabolites were identified between the BAP and HAP groups. Further screening out the metabolites of fold change values ≥ 2 found that phenylalanylhistidine was upregulated and benzyl sulfate was downregulated in the BAP group (*P* < 0.05).

### 3.4 Potential contribution of fecal markers of AP

**FIGURE 3:**
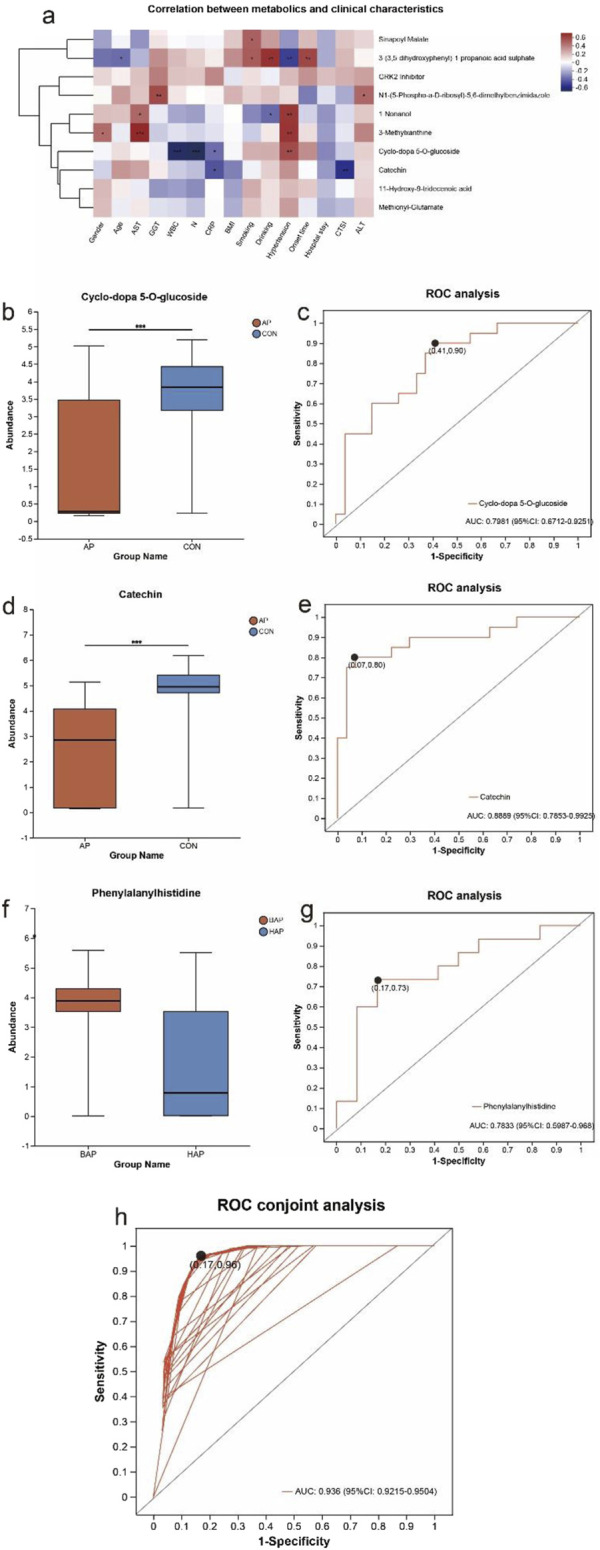
Diagnostic markers in feces for AP. **(a)** Pearson correlation between fecal differential metabolites and clinical characteristics linked with AP. The results were presented as a heatmap. Red squares indicate positive associations; blue squares indicate negative associations. *P < 0.05, **P < 0.01, ***P < 0.001. **(b)** Box-plot of expression of cyclo-dopa 5-O-glucoside. **(c)** Receiver operating characteristic curves for cyclo-dopa 5-O-glucoside. **(d)** Box-plot of expression of catechin. **(e)** Receiver operating characteristic curve for catechin. **(f)** Box-plot of expression of phenylalanylhistidine. **(g)** Receiver operating characteristic curve for phenylalanylhistidine. **(h)** Receiver operating characteristic curve for cyclo-dopa 5-O-glucoside and catechin.

Differential metabolites were identified by metabolites-expression-level analysis with *P* values ≤ 0.05 and fold change values ≥ 2, and VIP pred OPLS-DA>1. A total of 10 metabolites were included in the person correlation analysis. As shown in Figure 3a, significant correlations were observed between some fecal differential metabolites and clinical parameters including gender, age, BMI, CTSI score, and so on. A significant correlation of 3-(3,5-dihydroxyphenyl)-1-propanoic acid sulphate with the overall condition of the AP patients were observed, such as gender (r = −0.371, *P* = 0.057), age (r = −0.385, *P* = 0.048), history of smoking (r = 0.445, *P* = 0.020), history of drinking (r = 0.581, *P* = 0.001), hypertension (r = −0.514, *P* = 0.006). Besides, the degree of expression of 3-(3,5-dihydroxyphenyl)-1-propanoic acid sulphate was also involved with onset time (r = 0.513, *P* = 0.006). A significant positive correlation of N1-(5-Phospho-a-D-ribosyl)-5,6-dimethylbenzimidazole with alanine transaminase (r = 0.405, *P* = 0.036) and γ-Glutamyl Transferase (r = 0.500, *P* = 0.008) were observed. Besides, 3-Methylxanthine was involved with aspartate Transaminase (r = 0.616, *P* < 0.001). Cyclo-dopa 5-O-glucoside related to leukocyte (r = −0.628, *P* < 0.001), neutrophil ratio (r = −0.670, *P* < 0.001), and C-reactive protein (r = −0.383, *P* = 0.049). Catechin related to C-reactive protein (r = −0.455, *P* = 0.017) and CTSI score (r = −0.557, *P* = 0.003). The correlation tables were shown in Supplementary Tables S1, S2.

Compared with the control group, the expression level of Cyclo-dopa 5-O-glucoside related to inflammation index was significantly decreased in the AP group (Figure 3b). Receiver operating characteristic analysis found that Cyclo-dopa 5-O-glucoside was able to discriminate the AP patients from the controls with an AUC of 0.798 in these 50 subjects (Figure 3c). The expression level of catechin related to CTSI score was also significantly decreased in the AP group (Figure 3d). Receiver operating characteristic analysis found that catechin was able to discriminate the AP patients from the controls with an AUC of 0.889 in these 50 subjects (Figure 3e). Incorporating the above two metabolites into receiver operating characteristic conjoint analysis, it was found that Cyclo-dopa 5-O-glucoside and catechin were able to discriminate the AP patients from the controls with an AUC of 0.936 in these 50 subjects (Figure 3h).

As shown in Figure 3f, phenylalanylhistidine exhibited a considerable upregulation in the BAP group. Receiver operating characteristic analysis found that phenylalanylhistidine was able to discriminate BAP from HAP with an AUC of 0.783 in these 30 subjects (Figure 3g).

### 3.5 Significant differences in serum metabolites in patients with AP of different etiologies compared with controls

**FIGURE 4:**
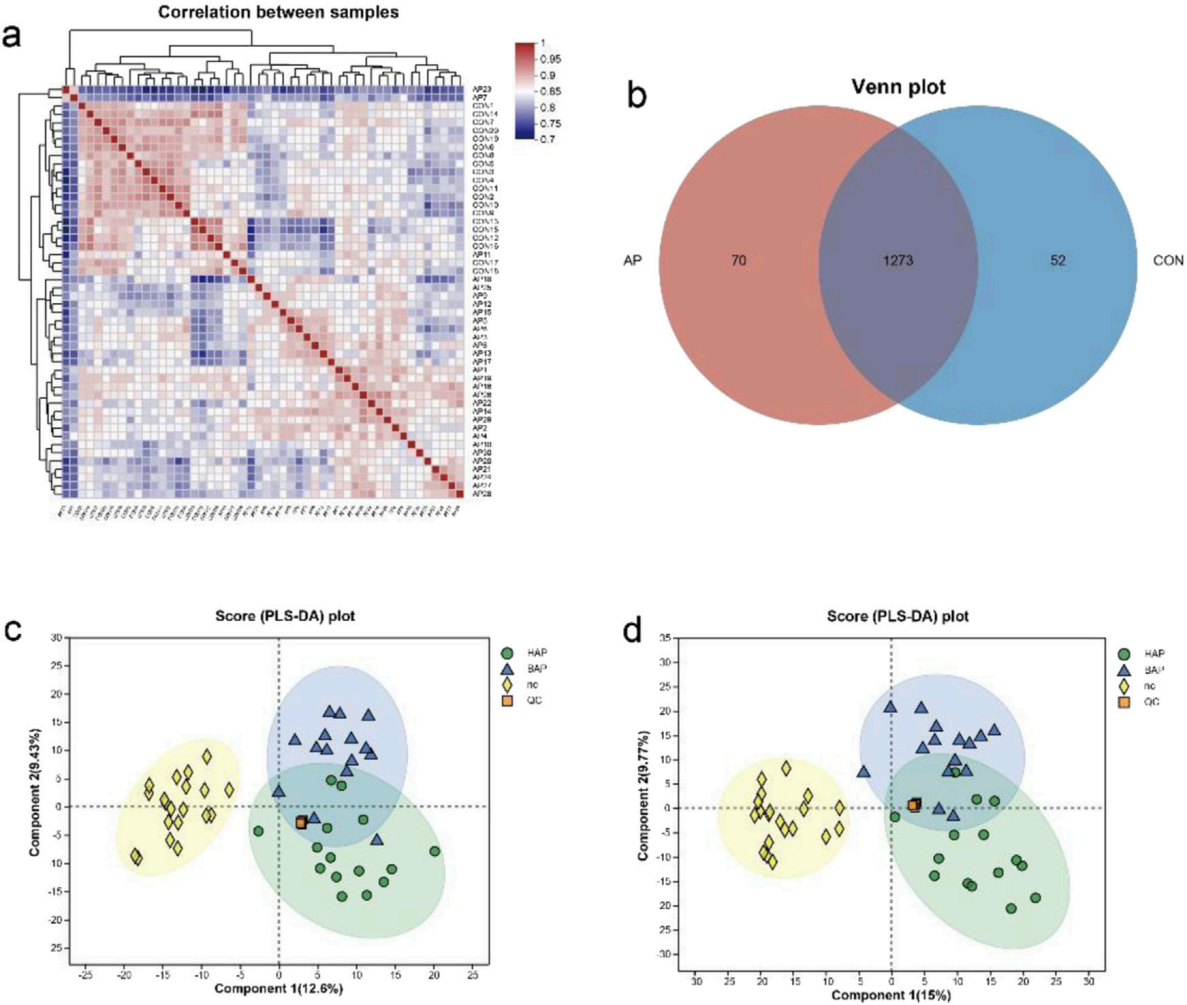
Differentiation of serum metabolome samples from patients with AP and controls. **(a)** Correlation analysis of serum samples between AP group and control group. **(b)** Venn analysis of serum metabolites between AP group and control group. **(c)** PLS-DA of serum metabolites in comparisons of patients with AP of different etiologies (positive ion). **(d)** PLS-DA of serum metabolites in comparisons of patients with AP of different etiologies (negative ion).

Pearson correlation analysis showed that the serum samples of AP group and control group were significantly separated (Figure 4a). As shown in Venn plot, 70 serum metabolites were unique in AP patients, and 52 serum metabolites were found in healthy people but not detected in AP patients (Figure 4b).

The separation between the AP and control groups could be clearly observed in the PLS-DA patterns in both negative- and positive-ion modes, which demonstrated that there was a severe metabolic disorder in the patients diagnosed with AP. Besides, the BAP group and the HAP group were also significantly different from the control group (Figures 4c,d). As shown in Supplementary Figure S2, all of the analyses in the OPLS-DA pattern were consistent with those in the PLS-DA pattern.

### 3.6 Differential metabolites and metabolic pathways in serum between patients with acute pancreatitis of different etiologies and control group

Differential metabolites were identified by metabolites-expression-level analysis with *P* values of ≤0.05 and fold change values ≥1.5, and VIP pred OPLS-DA>1. As shown in Table 6, 5 (3 upregulated and two downregulated) serum differential metabolites were identified between the AP and control groups. In order to conduct a more comprehensive analysis of the metabolic alterations in patients diagnosed with AP, an investigation of metabolic pathways was conducted. Among the five differential metabolites, the amount of L-Histidinol was much higher in the AP group, which is mainly involved in histidine metabolism (*P* < 0.05). The amount of stearidonic acid was substantially lower in the AP group, which is predominantly involved in alpha-Linolenic acid metabolism (*P* < 0.05).

**TABLE 6:** Differentially expressed serum metabolites between AP and CON groups.

Metabolite	Predicted molecular pathways or biological functions	AP versus CON			AP
		VIP	*P*	FC (AP/CON)	
ESI+					
Stearidonic acid	Lipid metabolism	3.4547	0.0016	0.5384	↓^5^
L-Histidinol	Histidine metabolism; Biosynthesis of amino acids	5.5623	<0.0001	1.5292	↑^2^
ESI-					
(2R,3R,4R,5S,6R)-2-(4-Chloro-3-(4-ethoxybenzyl)phenyl)-6-(hydroxymethyl)tetrahydro-2H-pyran-3,4,5-triol	-	3.4011	<0.0001	1.7485	↑^3^
4-Ethylphenylsulfate	-	3.6698	<0.0001	0.656	↓^4^
3-[4-(sulfooxy)phenyl]propanoic acid	-	6.2369	<0.0001	2.2189	↑^1^

^n^The order in which changes are evaluated based on VIP.

As shown in Table 7, 8 (4 upregulated and four downregulated) serum differential metabolites were identified between the BAP and control groups. The amount of caffeine was lower in the BAP group, which is involved in caffeine metabolism (0.05 < *P* < 0.1). In addition, same as the above, the amount of L-Histidinol was much higher in the BAP group, which is mainly involved in histidine metabolism (0.05 < *P* < 0.1). The amount of stearidonic acid was substantially lower in the BAP group, which is predominantly involved in alpha-Linolenic acid metabolism (0.05 < *P* < 0.1).

**TABLE 7:** Differentially expressed serum metabolites between BAP and CON groups.

Metabolite	Predicted molecular pathways or biological functions	BAP versus CON			BAP
		VIP	*P*	FC (AP/CON)	
ESI+					
Caffeine	Caffeine metabolism	4.2701	0.0002	0.6585	↓^7^
Stearidonic acid	alpha-Linolenic acid metabolism	3.9405	0.0017	0.4716	↓^8^
L-Histidinol	Histidine metabolism; Biosynthesis of amino acids	5.8176	<0.0001	1.6002	↑^2^
ESI-					
5-Methylpyrogallol sulfate	-	3.8256	<0.0001	0.6305	↓^5^
(2R,3R,4R,5S,6R)-2-(4-Chloro-3-(4-ethoxybenzyl)phenyl)-6-(hydroxymethyl)tetrahydro-2H-pyran-3,4,5-triol	-	3.8803	<0.0001	1.9256	↑^3^
5-Methoxynoracronycine	-	3.3549	<0.0001	1.5748	↑^4^
Sterebin D	-	3.3591	<0.0001	0.6299	↓^6^
3-[4-(sulfooxy)phenyl]propanoic acid	-	6.4660	<0.0001	2.3671	↑^1^

^n^The order in which changes are evaluated based on VIP.

As shown in Table 8, 6 (4 upregulated and two downregulated) fecal differential metabolites were identified between the HAP and control groups. The amount of stearidonic acid was substantially lower in the HAP group, which is predominantly involved in alpha-Linolenic acid metabolism (*P* < 0.05).

**TABLE 8:** Differentially expressed serum metabolites between HAP and CON groups.

Metabolite	Predicted molecular pathways or biological functions	HAP versus CON			HAP
		VIP	*P*	FC (AP/CON)	
ESI+					
Stearidonic acid	alpha-Linolenic acid metabolism	3.1882	0.0077	0.5456	↓^5^
Aspartyl-Arginine	-	2.7807	0.0280	1.9809	↑^6^
ESI-					
(2R,3R,4R,5S,6R)-2-(4-Chloro-3-(4-ethoxybenzyl)phenyl)-6-(hydroxymethyl)tetrahydro-2H-pyran-3,4,5-triol	-	2.8042	<0.0001	1.5742	↑^4^
7-Methyl-2′-deoxyguanosine-3′-monophosphate	-	3.8836	<0.0001	1.6278	↑^2^
4-Ethylphenylsulfate	-	3.7252	<0.0001	0.6218	↓^3^
3-[4-(sulfooxy)phenyl]propanoic acid	-	5.1823	<0.0001	2.0774	↑^1^

^n^The order in which changes are evaluated based on VIP.

Compared with the HAP group, aspartyl-Arginine was significantly decreased in the BAP group.

### 3.7 Potential contribution of serum markers of AP

**FIGURE 5:**
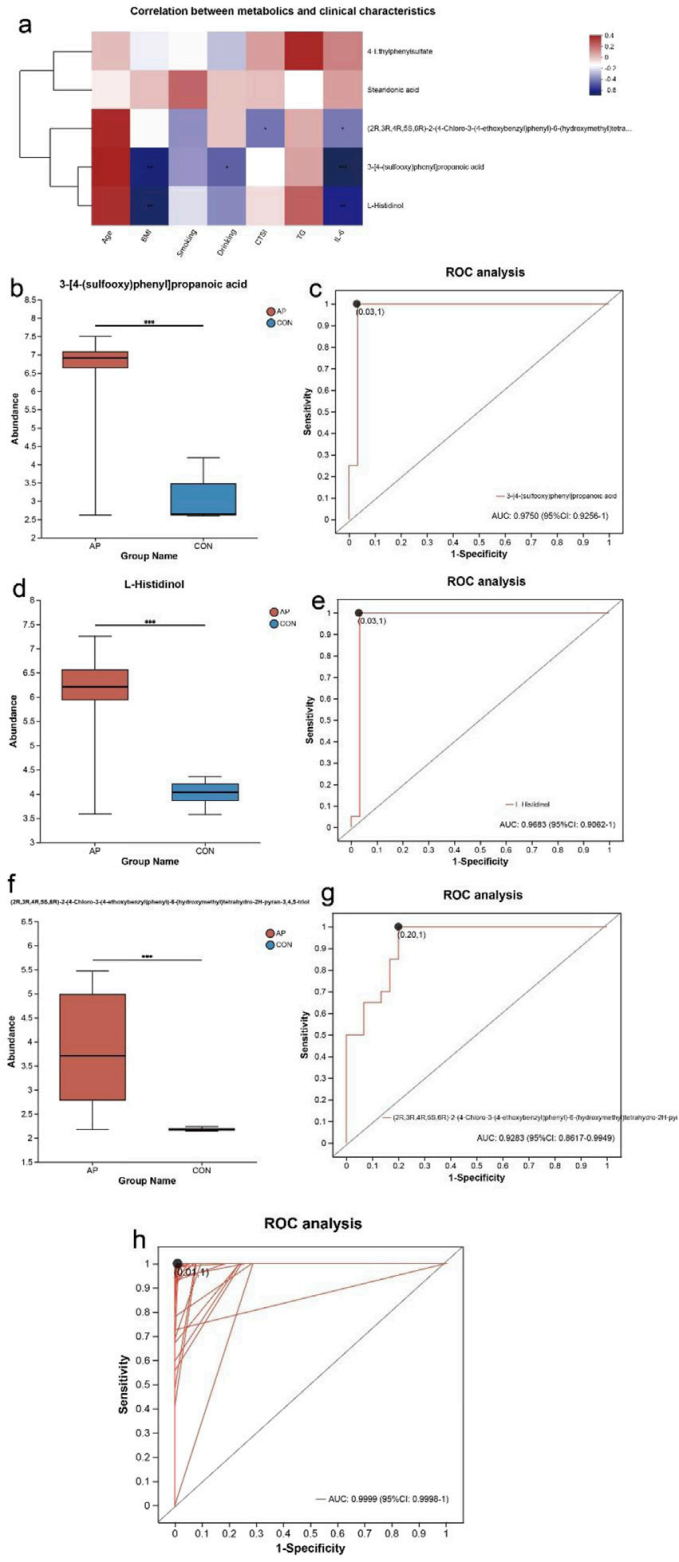
Diagnostic markers in serum for AP. **(a)** Pearson correlation between serum differential metabolites and clinical characteristics linked with AP. The results were presented as a heatmap. Red squares indicate positive associations; blue squares indicate negative associations. *P < 0.05, **P < 0.01, ***P < 0.001. **(b)** Box-plot of expression of 3-[4-(sulfooxy)phenyl]propanoic acid. **(c)** Receiver operating characteristic curves for 3-[4-(sulfooxy)phenyl]propanoic acid. **(d)** Box-plot of expression of L-Histidinol. **(e)** Receiver operating characteristic curves for L-Histidinol. **(f)** Box-plot of expression of (2R,3R,4R,5S,6R)-2-(4-Chloro-3-(4-ethoxybenzyl)phenyl)-6-(hydroxymethyl)tetrahydro-2H-pyran-3,4,5-triol. **(g)** Receiver operating characteristic curves for (2R,3R,4R,5S,6R)-2-(4-Chloro-3-(4-ethoxybenzyl)phenyl)-6-(hydroxymethyl)tetrahydro-2H-pyran-3,4,5-triol. **(h)** Receiver operating characteristic curves for 3-[4-(sulfooxy)phenyl]propanoic acid, L-Histidinol, and (2R,3R,4R,5S,6R)-2-(4-Chloro-3-(4-ethoxybenzyl)phenyl)-6-(hydroxymethyl)tetrahydro-2H-pyran-3,4,5-triol.

Differential metabolites were identified by metabolites-expression-level analysis with *P* values of ≤0.05 and fold change values ≥ 1.5, and VIP pred OPLS-DA>1. A total of five metabolites were included in the person correlation analysis. As shown in Figure 5a, noteworthy correlations were observed between some serum differential metabolites and clinical indicators including age, BMI, CTSI score, and so on. A significant correlation of 3-[4-(sulfooxy)phenyl]propanoic acid (r = −0.551, *P* = 0.002) and L-Histidinol (r = −0.569, *P* = 0.001) with BMI were observed. Both of 3-[4-(sulfooxy)phenyl]propanoic acid (r = −0.605, *P* < 0.001) and L-Histidinol (r = −0.535, *P* = 0.002) were also related with IL-6, which reflected to systemic inflammation of patients. Besides, the degree of expression of (2R,3R,4R,5S,6R)-2-(4-Chloro-3-(4-ethoxybenzyl)phenyl)-6-(hydroxymethyl)tetrahydro-2H-pyran-3,4,5-triol related with CTSI score (r = −0.371, *P* = 0.044) and IL-6 (r = −0.368, *P* = 0.046). The correlation tables were shown in Supplementary Tables S3, S4.

Compared with the control group, all of the expression level of 3-[4-(sulfooxy)phenyl]propanoic acid, L-Histidinol and (2R,3R,4R,5S,6R)-2-(4-Chloro-3-(4-ethoxybenzyl)phenyl)-6-(hydroxymethyl)tetrahydro-2H-pyran-3,4,5-triol were significantly increased in patients diagnosed with AP (Figures 5b,d,f). Receiver operating characteristic analysis found that 3-[4-(sulfooxy)phenyl]propanoic acid was able to discriminate the AP patients from the controls with an AUC of 0.975 in these 50 subjects (Figure 5c). L-Histidinol was able to discriminate the AP patients from the controls with an AUC of 0.968 in these 50 subjects (Figure 5e). (2R,3R,4R,5S,6R)-2-(4-Chloro-3-(4-ethoxybenzyl)phenyl)-6-(hydroxymethyl)tetrahydro-2H-pyran-3,4,5-triol was able to discriminate the AP patients from controls with an AUC of 0.928 in these 50 subjects (Figure 5g). Incorporating the above three metabolites into receiver operating characteristic conjoint analysis found that 3-[4-(sulfooxy)phenyl]propanoic acid, L-Histidinol and (2R,3R,4R,5S,6R)-2-(4-Chloro-3-(4-ethoxybenzyl)phenyl)-6-(hydroxymethyl)tetrahydro-2H-pyran-3,4,5-triol were able to discriminate the AP patients from the controls with an AUC of 0.999 in these 50 subjects (Figure 5j).

As shown in Figure 5h, Aspartyl-Arginine was downregulated significantly in the BAP group. Receiver operating characteristic analysis found that aspartyl-arginine in serum was able to discriminate the BAP patients from the HAP patients with an AUC of 0.724 in these 30 subjects (Figure 5i).

### 3.8 Conjoint analysis of fecal metabolites and serum metabolites

**FIGURE 6:**
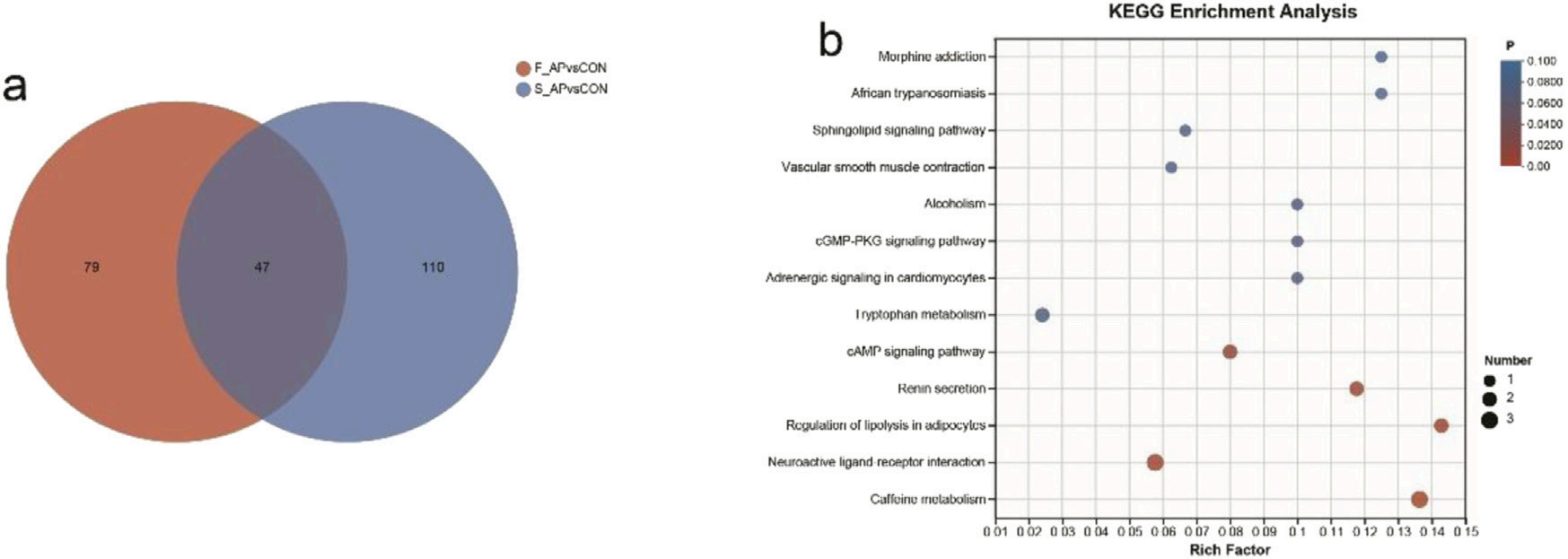
Conjoint analysis of fecal metabolites and serum metabolites. **(a)** Venn diagram of differential metabolites in serum and feces. **(b)** Metabolic pathway analysis on the differential metabolites found in both the serum and feces of the patients diagnosed with AP.

Differential metabolites were identified by metabolites-expression-level analysis with *P* values of ≤0.05 and fold change values ≥1, and VIP pred OPLS-DA>1. As shown in Figure 6a, a total of 126 differential metabolites in feces were identified between the AP and control groups. And there were 157 divergent metabolites in serum between the AP and control groups. A comprehensive analysis revealed the presence of 47 differential metabolites in both feces and serum samples. Among these metabolites, 26 exhibited an upregulation, while 21 displayed a downregulation. The 47 metabolites had significant associations with many biological processes, including caffeine metabolism, neuroactive ligand-receptor interaction, regulation of lipolysis in adipocytes, renin secretion, and cAMP signaling pathway (*P* < 0.05) (Figure 6b). The primary metabolites implicated in the metabolism of caffeine were found to be caffeine (decreased), paraxanthine (decreased), and theobromine (decreased). The differential metabolites that primarily participate in the neuroactive ligand-receptor interaction are adenosine (decreased), epinephrine (increased), and endomorphin-1 (increased). Besides, adenosine and epinephrine have been found to be involved in several metabolic pathways, such as renin secretion, the cAMP signaling system, and the regulation of lipolysis in adipocytes.

### 3.9 Safety and anti-inflammatory effects of gibberellin A4 and catechin

Subsequent characterization of the 47 candidate metabolites revealed distinct pathophysiological patterns. Specifically, fecal catechin concentrations in AP patients exhibited significant negative correlations with CTSI and CRP levels, paralleled by diminished serum catechin metabolites. Similarly, fecal Gibberellin A4 content demonstrated an inverse relationship with WBC and neutrophil percentage, accompanied by concurrent reduction of GA4-derived metabolites in systemic circulation. Consequently, Gibberellin A4 and catechin were specifically chosen to systematically assess their anti-inflammatory properties in pancreatic acinar cells.

**FIGURE 7:**
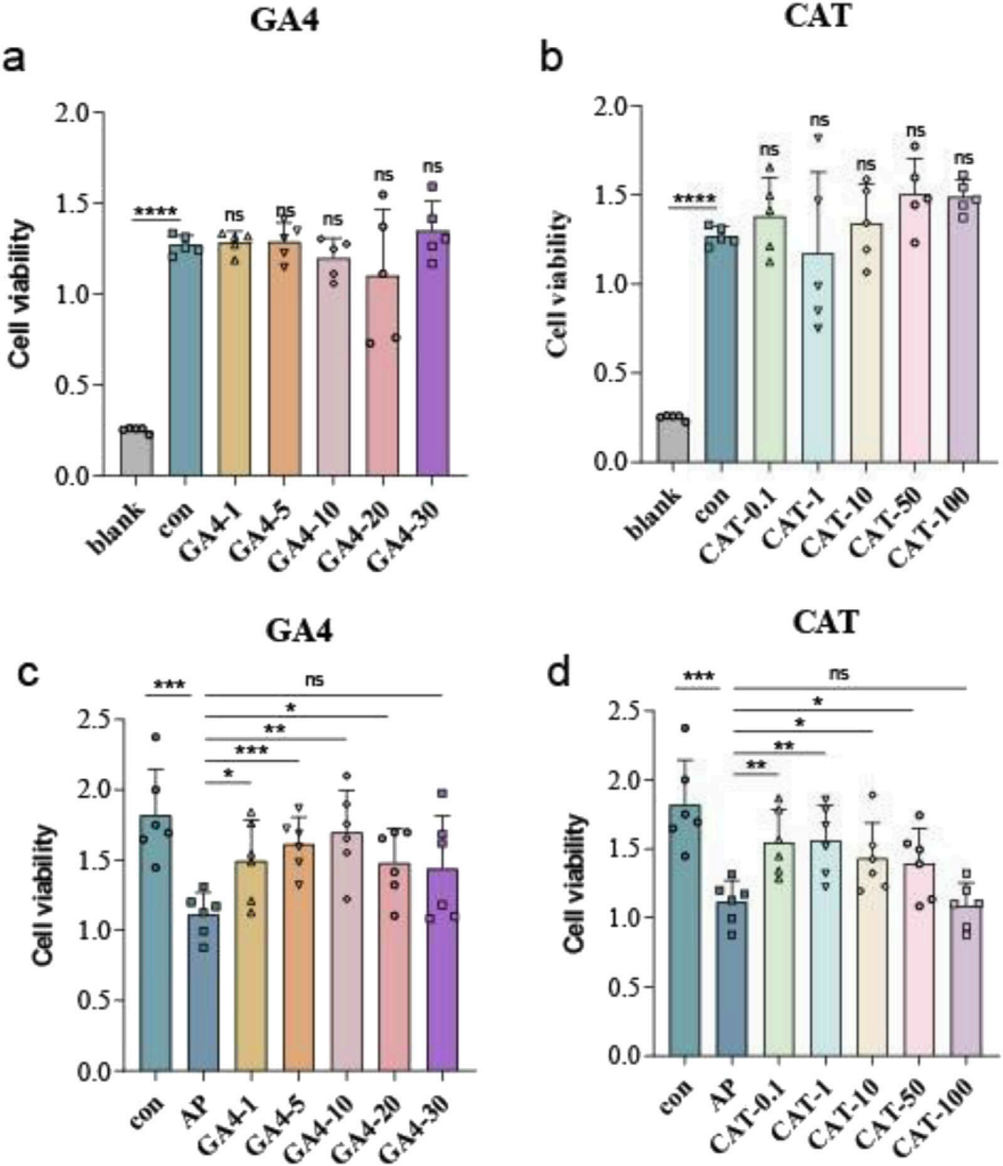
Cell activity with or without GA4 and catechin. **(a)** The effect of GA4 on cell viability was measured by CCK-8 assay. **(b)** The effect of catechin on cell viability was measured by CCK-8 assay. **(c)** Cell activity in different concentrations of GA4 in AP cells. **(d)** Cell activity in different concentrations of catechin in AP cells. *means P < 0.05, **means P < 0.01, ***means P < 0.001, ns means P > 0.05.

To assess potential cytotoxic effects, *in vitro* models were exposed to physiologically relevant concentrations of GA4 and catechin. Cytotoxicity assessment through CCK-8 assays revealed no statistically significant differences in cellular viability between treatment groups and vehicle controls across tested dosages (Figures 7a,b), confirming the biocompatibility of these compounds at investigated concentrations.

To systematically evaluate the cytoprotective and anti-inflammatory properties of GA4 and catechin, we subjected 266–6 pancreatic acinar cells to LPS (1 μg/mL, 24 h)-induced injury modeling acute pancreatitis (AP), with concomitant administration of escalating concentrations (GA4: 1–30 μM; catechin: 0.1–100 μM). Quantitative analysis through CCK-8 assays demonstrated concentration-dependent amelioration of cellular viability. GA4 exhibited maximal protection at 10 μM, while catechin showed optimal efficacy at 1 μM, establishing distinct effective concentration thresholds for the two compounds (Figures 7c,d).

**FIGURE 8:**
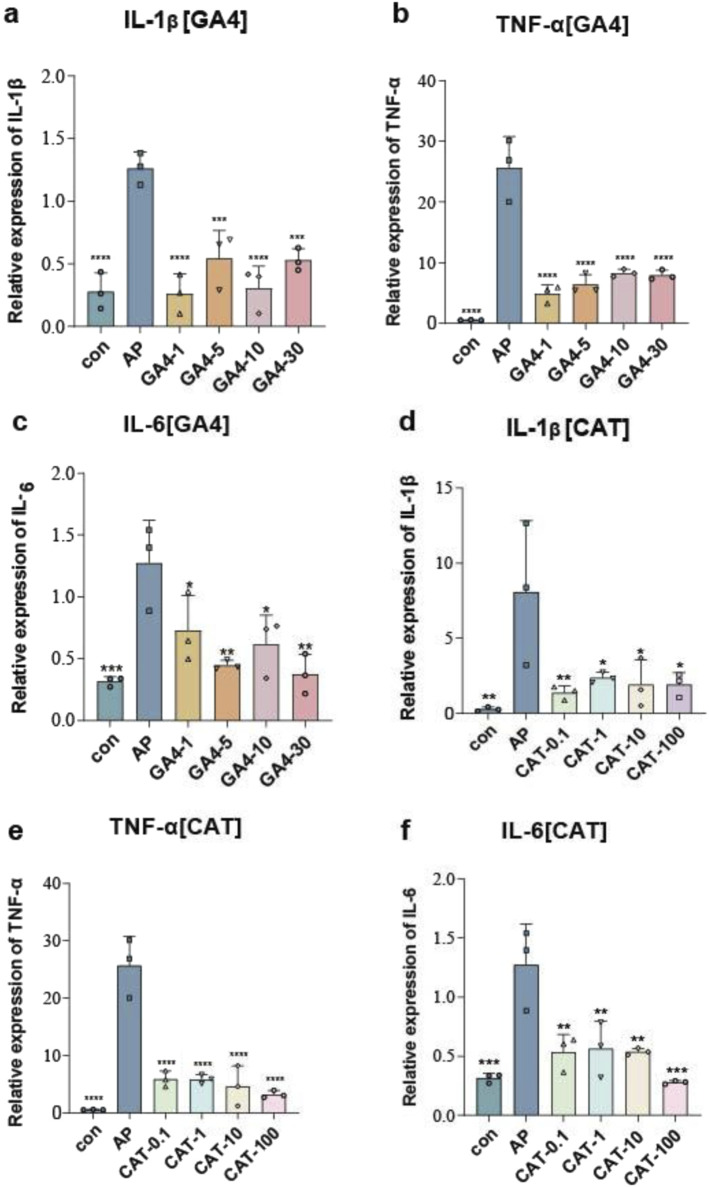
Impacts of GA4 and catechin on inflammatory factors. **(a–c)** The mRNA expression levels of IL-1β, TNF-α, and IL-6 in different concentrations of GA4. **(d–f)** The mRNA expression levels of IL-1β, TNF-α, and IL-6 in different concentrations of catechin. The symbol *means P < 0.05, **means P < 0.01, ***means P < 0.001 and ****means P < 0.0001, compared with AP.

Mechanistically, co-treatment with GA4 or catechin during LPS challenge resulted in marked attenuation of pro-inflammatory cytokine secretion. PCR quantification revealed that GA4 suppressed IL-1β, TNF-α and IL-6 production by 75.7%, 67.6% and 51.7% at 100 μM respectively (vs. LPS, *P* < 0.01). Catechin displayed dose-responsive inhibition patterns, achieving maximal anti-inflammatory effects at 100 μM with 76.2% (IL-1β), 87.5% (TNF-α) and 78% (IL-6) reductions (vs. LPS, *P* < 0.01) (Figure 8).

## 4 Discussion

In our research, we explored the fecal and serum metabolic changes of AP with different etiologies. And, we explored the correlation of the relevant differential metabolites with clinical indicators. A few studies have examined metabolites in patients or in animal models with AP. However, the majority of the studies have mostly concentrated on serum metabolites. There were no reports about fecal metabolites in AP patients in previous studies. Few studies have investigated the changes in metabolites in AP patients with different etiologies. This is the first study to reveal the changes in metabolites in AP patients with different etiology by using both fecal and serum metabolomics based on LC-MS.

In our study, between the patients diagnosed with AP and the controls, no statistically significant differences were seen in the majority of baseline conditions. However, there was a distinct trend of disparity in diabetes prevalence. In contrast to the patients diagnosed with BAP, patients diagnosed with HAP exhibited a higher prevalence of diabetes. In previous studies, metabolomic researches in diabetic patients, it was found that the levels of three branched-chain amino acids (i.e., leucine, isoleucine and valine) in plasma of diabetic patients were higher than those of normal people, and the level of branched-chain amino acids was correlated with insulin resistance. (Savolainen et al., 2017; Macotela et al., 2011; Newgard, 2012). The data of our research was consistent with previous studies. It was observed that the serum of the patients diagnosed with AP exhibited a modest elevation in the level of the three branched-chain amino acids. The FC value, however, was only about 1.1, indicating that the difference was not substantial. Differences of the level of branched-chain amino acids between the BAP and HAP groups were also the same as above. The findings of the study revealed that while there was a variation in the prevalence of diabetes among the groups, it had minimal impact on the comparison of metabolites among the different groups.

A notable disparity was seen in the fecal and serum metabolites between the patients diagnosed with AP and the controls. And the observed disparities in fecal and serum metabolites between patients diagnosed with BAP or HAP and the controls were also found to be statistically significant. There were no significant differences observed in the fecal metabolites between the BAP and HAP groups; nevertheless, notable differences were observed in the serum metabolome. This observation may be attributed to the resemblance in alterations in gut microbiota and the gut microbiota-associated metabolic alterations caused by different etiologies. However, differences in serum metabolites indicated that the metabolism in the bodies of patients with AP of different etiologies exhibits distinct variations.

We discovered that the levels of Gibberellin A7 (GA7) and catechin were significantly decreased in feces of patients with AP. Besides, serum concentrations of associated metabolites showed proportional declines. *In vitro* experiments demonstrated that these two metabolites were non-toxic and can significantly alleviate LPS-induced cell inflammation.

Catechin is classified as a phenolic compound, belonging to the flavonoid group, which is predominantly present in many plant species (Bansal et al., 2013). The primary source of catechin in the human body is derived from dietary intake. Upon entering the body, the substance undergoes hepatic metabolism, resulting in the production of metabolites that share a similar chemical structure, such as epicatechin (Latos-Brozio and Masek, 2020). The catechin contains phenolic groups and ester groups, which have been observed to interact with the Kelch-like ECH-associated protein 1 (Keap1) and stimulate the activation of the nuclear factor erythroid 2-related factor 2 (Nrf2) through the antioxidant response element. And increase the gene expression of heme oxygenase 1 (HO-1) or antioxidant enzymes, which can reduce inflammation and enhance the antioxidant property (Baranwal et al., 2022; Xie et al., 2020; Kim et al., 2022). Therefore, catechin exerts a protective effect on the human body by modulating the antioxidant response pathway. In addition, several experimental studies have indicated that catechin can inhibit the infiltration of neutrophils and the production of cytokines mediated by inflammasomes, thereby exerting a protective effect on the body (Zhao et al., 2022; Kim-Park et al., 2016). In summary, catechin serves as a protective component within the human body. Our research has preliminarily demonstrated that Catechin exert a protective effect on pancreatic cells and can inhibit the expression of inflammatory cytokines during AP, providing new insights into the treatment of AP.

Gibberellins (GAs) represent a large group of tetracyclic diterpenoid carboxylic acids with extensive structural variation. To date, more than 130 GAs structures have been identified. GAs are implicated in diverse biological processes, particularly in plants (Wang and Wang, 2022). However, the biological effects of GAs in mammals remain poorly understood. Reihill et al. reported a significant reduction in the release of interleukin 6 (IL-6) and interleukin 8 (IL-8) in primary nasal epithelial cells incubated with Gibberellic acid (GA3). They proposed that GA3 attenuates inflammation in airway epithelial cells, at least in part, through its impact on the nuclear factor kappa - B (NF-κB) and inhibitor of kappa-B alpha (IκBα) signaling pathways (Reihill et al., 2016). Nani et al. investigated the *in vitro* anti-NF-κB, anti-Candida, and antioxidant activities of Gibberellin A4 (GA4) and A7 (GA7). Their results showed that GA4 significantly inhibited NF-κB activation, while GA7 exhibited anti-Candida activity against *Candida* albicans (Nani et al., 2022). In this study, we examined the *in vitro* biological effects of GA4 and found it possessed anti - anti-inflammatory activity, suggesting its potential as an anti-inflammatory agent.

The KEGG pathway enrichment analysis conducted on fecal and serum metabolites derived from the BAP or HAP group revealed that both groups exhibited significant involvement in the metabolism of various amino acids and caffeine metabolism.

Caffeine metabolism in the AP patients, notably in the BAP patients, decreased significantly. Previous experimental research has demonstrated that caffeine has the ability to decrease abnormal calcium signals generated by pancreatic acinar cells in patients diagnosed with AP (Petersen and Sutton, 2006). In another experiment, it was shown that this protective effect may be achieved through inhibition of inositol 1,4,5-trisphosphate receptor (IP (3)R)-mediated signaling (Huang et al., 2017). In addition, in a meta-analysis including four studies with 351,137 participants found that heavy coffee drinkers have significantly lower risk of AP than non-coffee drinkers (Wijarnpreecha et al., 2018). The findings of all the studies indicated that caffeine has the potential to decrease inflammatory levels and mitigate the risk of AP. The findings presented are also consistent with the observed variability in caffeine levels within our research investigation. The potential correlation between the decrease in caffeine metabolism among the patients diagnosed with AP and the onset and progression of inflammation suggests a reciprocal causation relationship.

Although we explored the fecal and serum metabolic changes in AP with different etiologies, there are several limitations in this study. First, our sample size was constrained due to the exclusive reliance on a single hospital as the source of our samples. Further multi-center studies with larger sample sizes are needed in the future. Second, our study only utilized untargeted metabolomics technology based on LC-MS to analyze the samples. It is of great importance to use targeted metabolomics technology for the quantitative analysis of significant differential metabolites in the future.

In conclusion, there were significant differences in the fecal and serum metabolites and metabolic pathways of the AP group with different etiologies. These differential metabolites were correlated with several significant clinical indicators. The results of this study will help to further explore the pathogenesis of AP with different etiologies.

## Data Availability

The data generated in this study have been deposited in the MetaboLights repository (Yurekten et al., 2024) (https://www.ebi.ac.uk/metabolights) under accession numbers MTBLS8711 (https://www.ebi.ac.uk/metabolights/MTBLS8711) and MTBLS8766 (https://www.ebi.ac.uk/metabolights/MTBLS8766).
