# Supplementary material for: Explore the changes of metabolites in feces and serum of acute pancreatitis patients with different etiologies by LC-MS based metabolomics strategy
Source: Front Pharmacol. 2025 Jun 25;16:1614713. doi: 10.3389/fphar.2025.1614713 (PMC12237663; doi:10.3389/fphar.2025.1614713)
Supplement: Supplementary file 1 [file DataSheet1.zip › supplementary materials/supplement figure1.docx]

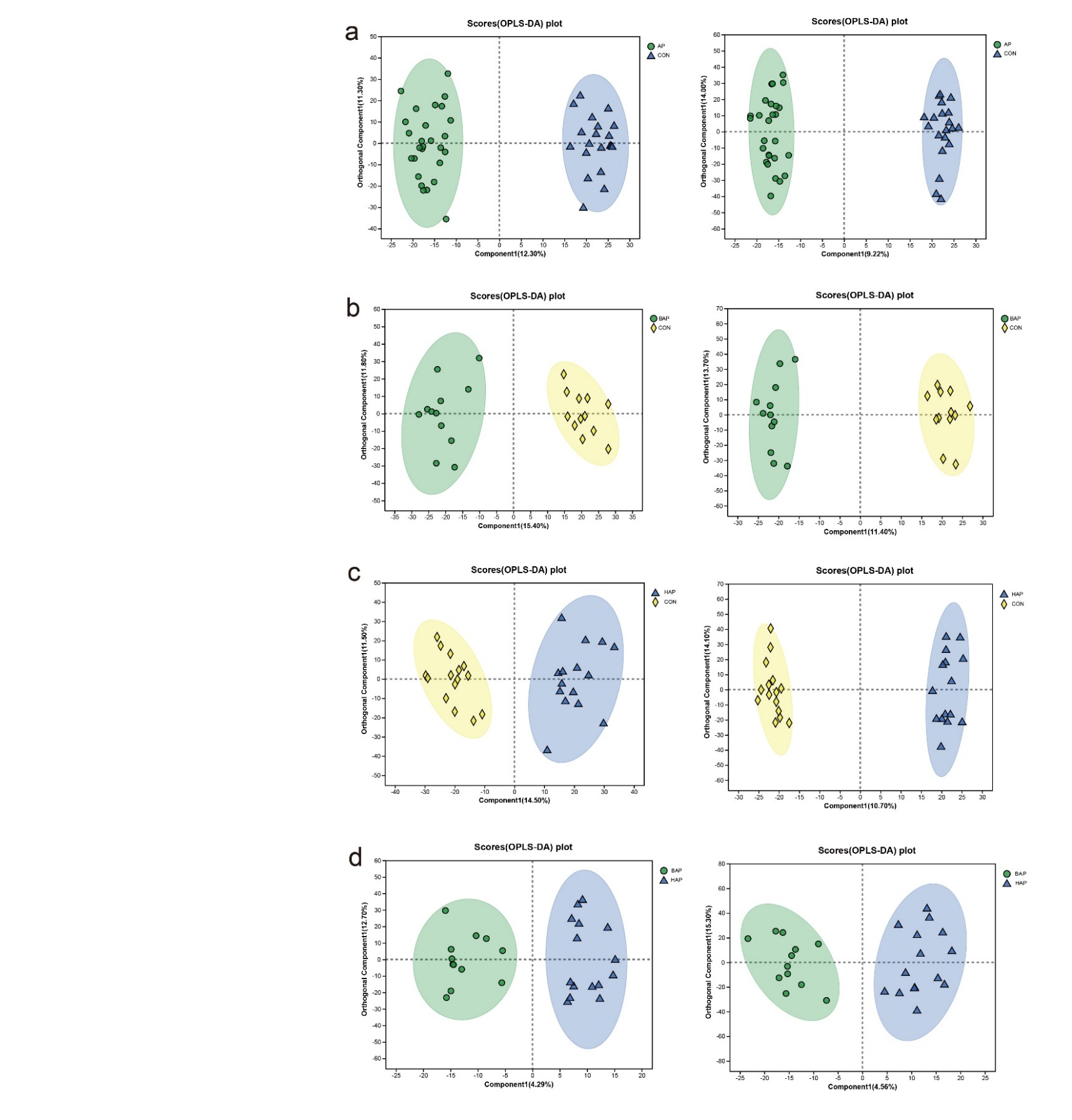


**Supplement Figure 1. Multivariate statistical analysis of fecal metabolomics of patients with acute pancreatitis. (a)** Orthogonal partial least squares discriminant analysis (OPLS-DA) of fecal metabolites in comparisons of the AP and control groups. **(b)** OPLS-DA of fecal metabolites in comparisons of the BAP and control groups. **(c)** OPLS-DA of fecal metabolites in comparisons of the HAP and control groups. **(d)** OPLS-DA of fecal metabolites in comparisons of the BAP and HAP groups. (Left: positive ion; right: negative ion)
