# Supplementary material for: Explore the changes of metabolites in feces and serum of acute pancreatitis patients with different etiologies by LC-MS based metabolomics strategy
Source: Front Pharmacol. 2025 Jun 25;16:1614713. doi: 10.3389/fphar.2025.1614713 (PMC12237663; doi:10.3389/fphar.2025.1614713)
Supplement: Supplementary file 1 [file DataSheet1.zip › supplementary materials/Table S1.docx]

| Metabolite | Gender | Age | AST | GGT | WBC | N | CRP | BMI | Smoking | Drinking | hypertension | Onset time | hospital stay | ALT | CTSI |
| --- | --- | --- | --- | --- | --- | --- | --- | --- | --- | --- | --- | --- | --- | --- | --- |
| Sinapoyl Malate | -0.2535 | -0.0544 | -0.2887 | 0.0499 | -0.0373 | -0.0545 | -0.0449 | 0.1227 | 0.4338 | 0.2471 | -0.2817 | 0.1624 | -0.2258 | 0.0901 | 0.1287 |
| 3-(3,5-dihydroxyphenyl)-1-propanoic acid sulphate | -0.3712 | -0.3847 | -0.2068 | 0.2929 | 0.1585 | 0.1015 | 0.0233 | 0.2924 | 0.445 | 0.581 | -0.5139 | 0.5132 | -0.2273 | 0.0009 | 0.1681 |
| GRK2 Inhibitor | 0.0899 | 0.0187 | 0.143 | 0.3398 | 0.1359 | 0.1311 | 0.3094 | -0.0888 | 0.0596 | -0.0613 | 0.1588 | 0.2732 | 0.1501 | 0.2353 | 0.1883 |
| N1-(5-Phospho-a-D-ribosyl)-5,6-dimethylbenzimidazole | -0.0054 | 0.252 | 0.1327 | 0.4997 | 0.0431 | -0.0249 | -0.0008 | 0.1827 | 0.0942 | 0.1419 | 0.07 | -0.3456 | -0.1012 | 0.4053 | -0.2252 |
| 1-Nonanol | 0.1525 | 0.1407 | 0.4065 | -0.0772 | -0.2379 | -0.2611 | -0.0777 | -0.0237 | -0.3138 | -0.3933 | 0.5623 | -0.1325 | -0.2888 | 0.124 | -0.1116 |
| 3-Methylxanthine | 0.3939 | -0.0762 | 0.6158 | -0.1523 | -0.0975 | -0.1289 | -0.096 | -0.0401 | -0.1496 | 0.0339 | 0.5717 | 0.063 | -0.2661 | 0.3181 | -0.086 |
| Cyclo-dopa 5-O-glucoside | -0.0487 | 0.062 | -0.0004 | -0.069 | -0.6275 | -0.6702 | -0.383 | -0.046 | 0.1898 | 0.1969 | 0.4981 | 0.2926 | -0.3164 | 0.0998 | -0.1484 |
| Catechin | -0.1357 | 0.2672 | 0.2259 | 0.0257 | -0.1483 | -0.1812 | -0.4555 | -0.3315 | -0.0594 | 0.1105 | 0.2821 | -0.0376 | -0.2728 | 0.0775 | -0.5571 |
| 11-Hydroxy-9-tridecenoic acid | 0.1683 | 0.0264 | -0.07 | -0.2831 | -0.2626 | -0.3079 | -0.0263 | -0.2544 | 0.2009 | 0.0594 | 0.1643 | 0.1215 | -0.1817 | 0.0925 | -0.0421 |
| Methionyl-Glutamate | 0.1554 | -0.0679 | 0.0296 | -0.1015 | -0.1144 | -0.1783 | -0.0338 | -0.2199 | 0.1372 | 0.0559 | 0.2461 | -0.0773 | -0.218 | 0.1173 | -0.1867 |

Supplementary Table 1. The correlation of fecal metabolites with clinical parameters (R value)
