# Supplementary material for: Explore the changes of metabolites in feces and serum of acute pancreatitis patients with different etiologies by LC-MS based metabolomics strategy
Source: Front Pharmacol. 2025 Jun 25;16:1614713. doi: 10.3389/fphar.2025.1614713 (PMC12237663; doi:10.3389/fphar.2025.1614713)
Supplement: Supplementary file 1 [file DataSheet1.zip › supplementary materials/Table S2.docx]

Supplementary Table 2. The correlation of metabolites with clinical parameters (*p*-value)

| Metabolite | Gender | Age | AST | GGT | WBC | N | CRP | BMI | Smoking | Drinking | Hypertension | Onset time | Hospital stay | CTSI | ALT |
| --- | --- | --- | --- | --- | --- | --- | --- | --- | --- | --- | --- | --- | --- | --- | --- |
| Sinapoyl Malate | 0.20198 | 0.78761 | 0.14416 | 0.80486 | 0.85352 | 0.78723 | 0.82419 | 0.54215 | 0.02379 | 0.21394 | 0.15452 | 0.4182 | 0.25752 | 0.52225 | 0.65493 |
| 3-(3,5-dihydroxyphenyl)-1-propanoic acid sulphate | 0.05663 | 0.04753 | 0.3007 | 0.13817 | 0.42982 | 0.61444 | 0.90811 | 0.13893 | 0.02002 | 0.00148 | 0.00611 | 0.00619 | 0.25416 | 0.40183 | 0.9965 |
| GRK2 Inhibitor | 0.65578 | 0.92633 | 0.47671 | 0.08289 | 0.49913 | 0.51452 | 0.11626 | 0.65969 | 0.76768 | 0.76129 | 0.42893 | 0.16798 | 0.45495 | 0.34682 | 0.23737 |
| N1-(5-Phospho-a-D-ribosyl)-5,6-dimethylbenzimidazole | 0.97857 | 0.2048 | 0.50942 | 0.00796 | 0.83104 | 0.90196 | 0.99689 | 0.36173 | 0.64035 | 0.48006 | 0.72865 | 0.07744 | 0.61539 | 0.25873 | 0.03599 |
| 1-Nonanol | 0.44763 | 0.48397 | 0.03537 | 0.70204 | 0.23203 | 0.18832 | 0.69995 | 0.90662 | 0.11091 | 0.04239 | 0.00227 | 0.51008 | 0.144 | 0.57929 | 0.53785 |
| 3-Methylxanthine | 0.04205 | 0.70558 | 0.00063 | 0.44817 | 0.62848 | 0.5217 | 0.6339 | 0.84246 | 0.45651 | 0.86674 | 0.00184 | 0.75509 | 0.17968 | 0.66962 | 0.10584 |
| Cyclo-dopa 5-O-glucoside | 0.80939 | 0.75868 | 0.99825 | 0.73247 | 0.00046 | 0.00013 | 0.04859 | 0.81985 | 0.34303 | 0.32491 | 0.00819 | 0.13852 | 0.10782 | 0.46015 | 0.62052 |
| Catechin | 0.49981 | 0.17793 | 0.25728 | 0.89888 | 0.46029 | 0.36562 | 0.01697 | 0.09121 | 0.76868 | 0.58324 | 0.15399 | 0.8521 | 0.16863 | 0.00254 | 0.70068 |
| 11-Hydroxy-9-tridecenoic acid | 0.40133 | 0.89584 | 0.72847 | 0.15245 | 0.18565 | 0.11822 | 0.89648 | 0.20032 | 0.31488 | 0.76841 | 0.41283 | 0.54611 | 0.36447 | 0.83468 | 0.64645 |
| Methionyl-Glutamate | 0.43888 | 0.73661 | 0.88358 | 0.61434 | 0.56982 | 0.37359 | 0.86721 | 0.27044 | 0.49503 | 0.78172 | 0.21598 | 0.70159 | 0.2747 | 0.35106 | 0.56014 |
