# Supplementary material for: Explore the changes of metabolites in feces and serum of acute pancreatitis patients with different etiologies by LC-MS based metabolomics strategy
Source: Front Pharmacol. 2025 Jun 25;16:1614713. doi: 10.3389/fphar.2025.1614713 (PMC12237663; doi:10.3389/fphar.2025.1614713)
Supplement: Supplementary file 1 [file DataSheet1.zip › supplementary materials/Table S3.docx]

Supplementary Table 3. The correlation of serum metabolites with clinical parameters (R value)

| Metabolite | Age | BMI | Smoking | Drinking | CTSI | TG | IL-6 |
| --- | --- | --- | --- | --- | --- | --- | --- |
| 4-Ethylphenylsulfate | 0.0103 | -0.162 | -0.1074 | -0.2369 | 0.075 | 0.3407 | 0.139 |
| Stearidonic acid | -0.0778 | 0.0076 | 0.2021 | -0.0034 | 0.0086 | -0.1304 | 0.076 |
| (2R,3R,4R,5S,6R)-2-(4-Chloro-3-(4-ethoxybenzyl)phenyl)-6-(hydroxymethyl)tetrahydro-2H-pyran-3,4,5-triol | 0.336 | -0.1095 | -0.3244 | -0.006 | -0.371 | 0.0508 | -0.3676 |
| 3-[4-(sulfooxy)phenyl]propanoic acid | 0.3556 | -0.551 | -0.3141 | -0.4114 | -0.1316 | 0.0692 | -0.6047 |
| L-Histidinol | 0.3247 | -0.5686 | -0.1845 | -0.3333 | -0.0539 | 0.2137 | -0.5347 |
