# Supplementary material for: Explore the changes of metabolites in feces and serum of acute pancreatitis patients with different etiologies by LC-MS based metabolomics strategy
Source: Front Pharmacol. 2025 Jun 25;16:1614713. doi: 10.3389/fphar.2025.1614713 (PMC12237663; doi:10.3389/fphar.2025.1614713)
Supplement: Supplementary file 1 [file DataSheet1.zip › supplementary materials/Table S4.docx]

Supplementary Table 4. The correlation of serum metabolites with clinical parameters (*p*-value)

| Metabolite | Age | BMI | Smoking | Drinking | CTSI | TG | IL-6 |
| --- | --- | --- | --- | --- | --- | --- | --- |
| 4-Ethylphenylsulfate | 0.95673 | 0.39242 | 0.57214 | 0.20742 | 0.69378 | 0.06542 | 0.46395 |
| Stearidonic acid | 0.68266 | 0.96823 | 0.28422 | 0.9857 | 0.96395 | 0.49236 | 0.68983 |
| (2R,3R,4R,5S,6R)-2-(4-Chloro-3-(4-ethoxybenzyl)phenyl)-6-(hydroxymethyl)tetrahydro-2H-pyran-3,4,5-triol | 0.06947 | 0.56452 | 0.08034 | 0.97489 | 0.04357 | 0.7896 | 0.04569 |
| 3-[4-(sulfooxy)phenyl]propanoic acid | 0.05382 | 0.0016 | 0.09097 | 0.0239 | 0.48803 | 0.71648 | 0.0004 |
| L-Histidinol | 0.08002 | 0.00104 | 0.32916 | 0.07191 | 0.77718 | 0.25687 | 0.00233 |
